# European training requirements in geriatric medicine 2025: driving competency-based education and harmonisation across Europe

**DOI:** 10.1007/s41999-025-01393-8

**Published:** 2026-02-17

**Authors:** Román Romero Ortuño, Marianne van Iersel, Maria S. Nuotio, Helgi Kolk, Eva Topinková, Didier Schoevaerdts, Maria Victoria Farré Mercadé, Christophe Graf, Santiago Cotobal Rodeles, Mark Anthony Vassallo, Michael Vassallo, Dieter Lütje, Jūratė Macijauskienė

**Affiliations:** 1 Discipline of Medical Gerontology, School of Medicine, Trinity College Dublin, Mercer’s Institute for Successful Ageing, St James’s Hospital, James’s Street, Dublin, D08 NHY1 Ireland; 2 Department of Geriatric Medicine, Radboud University, Medical Center, Nijmegen, The Netherlands; 3 Department of Geriatric Medicine, Faculty of Medicine, University of Turku, Turku University Hospital, Turku, Finland; 4 Faculty of Medicine, University of Tartu, Tartu University Hospital, Tartu, Estonia; 5 Department of Geriatrics, First Faculty of Medicine, Charles University and General University Hospital, Prague, Czech Republic; 6 Department of Geriatric Medicine, Centre Hospitalier Universitaire UCL Namur, Namur, Belgium; 7 Medicine Department, Universitat Internacional de Catalunya (UIC Barcelona), Barcelona, Spain; 8 Department of Readaptation and Geriatrics, Geneva University Hospitals, Geneva, Switzerland; 9 European Junior Doctors (EJD) representative to UEMS-GMS, Unidad de Geriatría, Complejo Asistencial Universitario, Salamanca, Spain; 10 Karin Grech Rehabilitation Hospital, Telghet G’Magia, Tal-Pieta’, Malta; 11 Royal Bournemouth Hospital, University Hospitals Dorset, Bournemouth, UK; 12 Medizinische Klinik IV, Geriatrie Und Palliativmedizin, Klinikum Osnabrück, Osnabrück, Germany; 13 Department of Geriatrics, Lithuanian University of Health Sciences, Kaunas, Lithuania; 14 Institute for Postgraduate Medical Education, Division of Geriatric Medicine, Prague, Czech Republic; 15 Faculty of Health and Social Sciences, University of South Bohemia, České Budejovice, Czech Republic; 16 UCLouvain, Institute of Health and Society, Brussels, Belgium; 17 Geriatric Medicine Department, Hospital General de Granollers, Barcelona, Spain; 18 Department of Readaptation and Geriatrics, University of Geneva, Geneva, Switzerland

**Keywords:** Geriatric medicine, Postgraduate education, Competency-based education, Entrustable professional activities, UEMS, European Training Requirements, Curriculum

## Abstract

**Purpose:**

To describe the process, content, and significance of the 2025 revision of the European Training Requirements (ETR) for the Specialty of Geriatric Medicine, developed under the auspices of the European Union of Medical Specialists-Geriatric Medicine Section (UEMS-GMS). The revision aims to update European postgraduate training standards to reflect current scientific, clinical, and educational advances.

**Process:**

The revision followed the official UEMS procedure for ETR development and was conducted by the UEMS-GMS ETR Review Committee between September 2024 and October 2025. Building on the 2019 European Postgraduate Curriculum in Geriatric Medicine and the 2020 ETR, the committee incorporated stakeholder feedback, expert consultation, and international endorsement. The final document was reviewed and formally approved by the UEMS-GMS and endorsed by the European Geriatric Medicine Society (EuGMS), the European Academy for Medicine of Ageing (EAMA), the International Association of Gerontology and Geriatrics (IAGG), and the European Interdisciplinary Council on Ageing (EICA).

**Highlights:**

The 2025 ETR retains the established UEMS three-part structure (for trainees, trainers, and training institutions) whilst introducing a modernised, competency-based education framework. The updated syllabus expands and refines theoretical content to reflect the latest scientific, clinical, and pedagogical advances. The revised ETR further elaborates the use of Entrustable Professional Activities (EPAs) as core instruments for assessing competence. Assessment standards now feature an expanded toolkit, incorporating the knowledge-based European Geriatric Medicine Specialty Exam (EGeMSE).

**Significance:**

The 2025 ETR for Geriatric Medicine represents a further step toward harmonised, competency-based specialist training across Europe and beyond. Reflecting global public health priorities, including the UN Decade of Healthy Ageing (2021–2030), the revision reinforces educational excellence and the delivery of high-quality, integrated, person-centred care for older adults. It also supports the continued development of the specialty of Geriatric Medicine in countries where it is not yet established or remains emerging. LINK to ETR 2025: https://www.uems.eu/european-training-requirements (Geriatric Medicine, 2025/29).

## Key summary points

**Aim:**

To present and contextualise the 2025 revision of the European Training Requirements (ETR) for the Specialty of Geriatric Medicine, developed under the auspices of the European Union of Medical Specialists-Geriatric Medicine Section (UEMS-GMS), and to summarise its main innovations in structure, content, and pedagogical approach.

**Highlights:**

The 2025 ETR strengthens the 2020 version by further developing Entrustable Professional Activities (EPAs) to harmonise postgraduate geriatric training across Europe. It updates and expands the content and recommends the knowledge-based European Geriatric Medicine Specialty Exam (EGeMSE) as part of the certification standards.

**Message:**

The 2025 ETR reflects the continued evolution of European geriatric medicine education, uniting scientific progress and competency-based pedagogy within a coherent, evidence-informed framework that promotes excellence, mobility, and comparability of specialist training across Europe. It establishes a forward-looking standard designed to remain fit for purpose over the next 5 years.

## Introduction

In response to the well-known and ongoing ageing of populations [1, 2], the World Health Organization (WHO) World Report on Ageing and Health (2015) [3] and the United Nations (UN) Decade of Healthy Ageing (2021–2030) [4] have called for comprehensive reform of health systems, workforce development, and education to promote equitable, integrated, and person-centred care for older people.

Within this evolving landscape, Geriatric Medicine is the medical specialty dedicated to the care of older adults across the continuum of acute, chronic, rehabilitative, preventive, and end-of-life settings. It focuses on individuals living with frailty and multimorbidity, whose needs extend beyond organ-based paradigms of care and demand multidisciplinary, holistic management aimed at preserving function, autonomy, and quality of life [5]. A geriatrician is defined as a medical doctor who specialises in the care of older people and who possesses the formal competencies to assess and manage the complex medical, psychological, functional, and social issues associated with ageing, including their broader social consequences. Their principal expertise lies in the implementation and delivery of Comprehensive Geriatric Assessment (CGA) across care settings, coordinating multidisciplinary inputs to optimise health outcomes, functional, cognitive, and psychological abilities, and quality of life for older adults [6]. CGA, a defining feature of the specialty, is a multidimensional, interdisciplinary diagnostic and therapeutic process with robust evidence for improving outcomes such as survival, recovery, and independence following acute illness [7, 8]. Thus, CGA represents the foundation of geriatric practice and a unifying educational principle for specialist training [9].

**Fig. 1:**
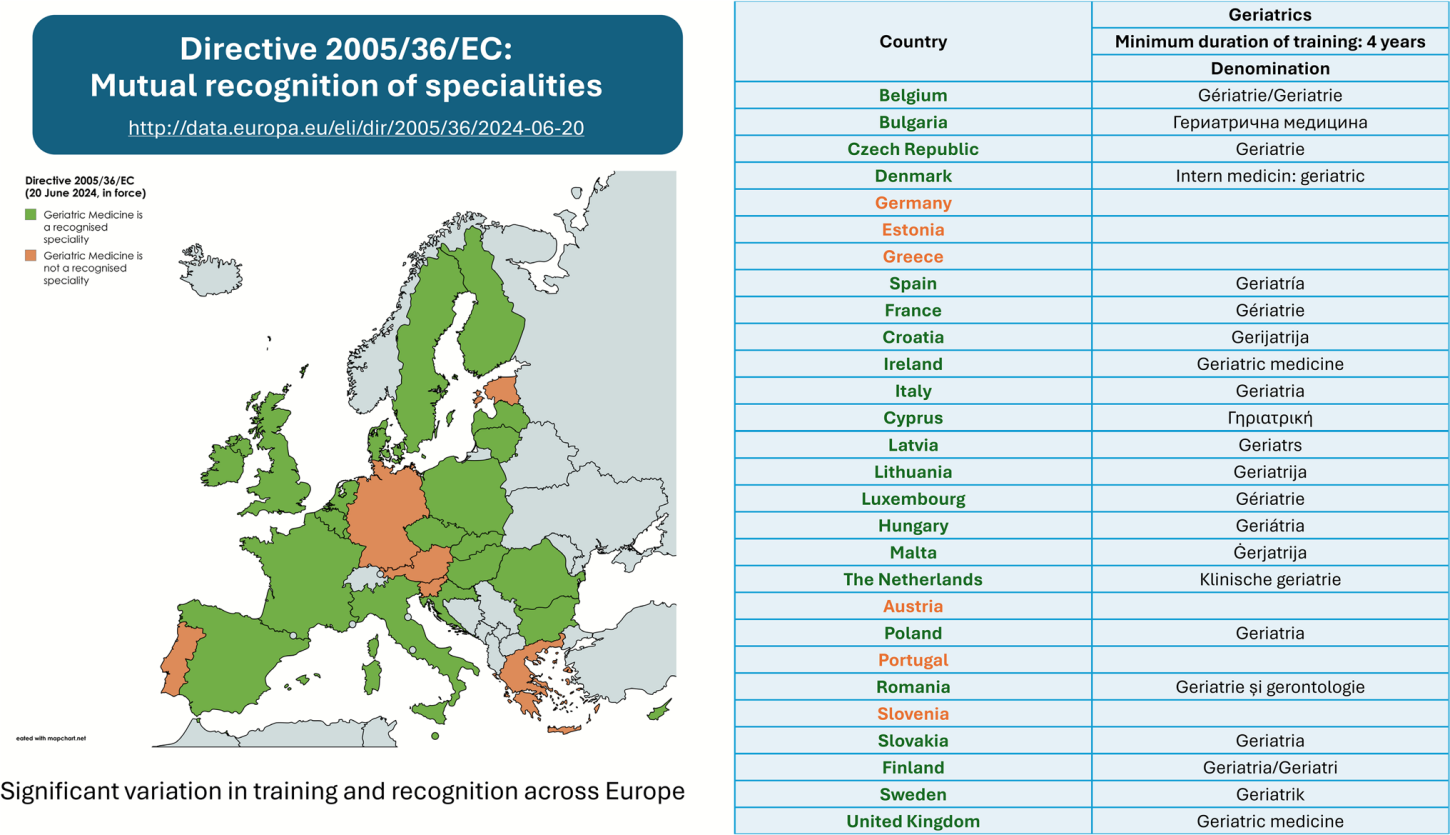
Recognition of Geriatric Medicine under Directive 2005/36/EC (June 2024). Green = recognised; orange = not explicitly recognised at country level; grey = no data/non-EU. Map created with mapchart.net

Despite this clear professional identity of Geriatric Medicine, the latest consolidated EU Directive on the recognition of professional qualifications (Directive 2005/36/EC, updated June 20, 2024) does not yet include Geriatric Medicine as a uniformly recognised primary specialty across all States [10] (Fig. 1). In several European countries, the discipline remains classified as a subspecialty, or absent altogether from national specialty registers, highlighting persistent heterogeneity in recognition and training frameworks [11]. This inconsistency underscores the strategic importance of establishing shared European standards that ensure comparability, quality, and professional mobility. By defining a harmonised set of competencies and learning outcomes, the European Training Requirements (ETR) contribute directly to the broader European agenda of equitable, high-quality care for ageing populations.

The ETRs are developed under the auspices of the Union Européenne des Médecins Spécialistes/European Union of Medical Specialists (UEMS), an international non-profit association (AISBL) founded in 1958. The UEMS is the oldest medical organisation in Europe and represents the national associations of medical specialists from 42 member countries. It provides expert advice to the European Commission and other EU institutions on issues related to postgraduate medical education, professional mobility, and healthcare quality [12]. Within the UEMS, each medical discipline is represented by a Section, which focuses on specialty-specific training and standards, and may be supported by a European Board responsible for coordinating examinations and quality assurance activities. Geriatric Medicine is represented through the UEMS Geriatric Medicine Section (UEMS-GMS) and Board, which jointly oversee the development and maintenance of European standards for specialist education, including the ETR and the newly piloted European Geriatric Medicine Specialty Exam (EGeMSE).

The ETRs define common frameworks and minimum standards for postgraduate specialist education, complementing national regulations whilst promoting harmonisation, excellence, and professional recognition across borders. They originate from Chapter 6 of the European Charter for Medical Training [13] and are updated approximately every 5 years to reflect scientific, clinical, and pedagogical advances. Each ETR establishes a set of minimum requirements that may be adapted to national contexts, ensuring both quality and flexibility across diverse healthcare systems. Developed by professionals, for professionals, the ETRs translate European directives into practice and serve as authoritative instruments for quality assurance in specialist training, though they do not constitute legal directives themselves.

The fundamental principles of the ETRs rest on the premise that the quality of medical care is directly proportional to the quality of medical training. Their pedagogical model is grounded in the CanMEDS framework, which defines the physician as a medical expert who integrates complementary roles as communicator, collaborator, leader, health advocate, scholar, and professional [14]. ETRs promote competency-based education and workplace-based assessment as key instruments to ensure consistency and excellence in specialist training throughout Europe. A central distinction within ETRs lies between knowledge (the theoretical and contextual understanding of essential medical concepts acquired through study, teaching, and clinical experience) and competence (the demonstrated ability to apply this knowledge, along with professional skills and attitudes, in real clinical contexts safely and autonomously). Knowledge is typically assessed through examinations (e.g. written, oral), whereas competence is evaluated through workplace-based methods including Entrustable Professional Activities (EPAs), which reflect the integrated performance expected of a fully trained specialist.

This Special Article aims to present and contextualise the 2025 ETR for the Specialty of Geriatric Medicine, developed under the auspices of the UEMS-GMS. The previous version of the ETR was formally approved by the UEMS Council on October 17, 2020. In the 5 years since its publication, advances in science, education, healthcare delivery, and policy have created the need for an updated framework that remains fit for purpose. Accordingly, the 2025 revision was undertaken by the UEMS-GMS through a structured and collaborative process to ensure that the document continues to reflect current best practice and the evolving needs of ageing populations across Europe.

This article outlines the background, guiding principles, and educational philosophy underpinning the 2025 revision. It highlights the ETR’s central role in promoting consistency, comparability, and excellence in specialist training, whilst situating it within the broader mission of the UEMS to harmonise postgraduate medical education, support professional mobility, and enhance the quality of healthcare for older adults.

Formally endorsed by the European Geriatric Medicine Society (EuGMS), the European Academy for Medicine of Ageing (EAMA), the International Association of Gerontology and Geriatrics (IAGG), and the European Interdisciplinary Council on Ageing (EICA), the 2025 ETR embodies a shared European commitment to advancing education in ageing medicine and ensuring the delivery of high-quality, person-centred care for Europe’s growing older population.

## Process

The revision process for the 2025 ETR for the Specialty of Geriatric Medicine followed a structured and transparent methodology under the auspices of the UEMS-GMS. It adhered to the official UEMS procedures and rules governing the development of ETRs [15] and was implemented from September 2024 to October 2025.

### Initiation and committee formation

The revision was formally launched at the UEMS-GMS in-person and online meeting held in Valencia, Spain, on September 21, 2024, following the annual EuGMS Congress. A UEMS-GMS ETR Review Committee was subsequently established, comprising national representatives from UEMS member countries and operating under the UEMS’ standard Terms of Reference [15]. The Committee membership is detailed in Table 1.

**Table 1:** Members of the UEMS-GMS ETR 2025 Review Committee and their roles and countries of representation

ETR 2025 review committee	Role/country of representation
Román Romero Ortuño	Chair of the ETR Review Committee (Ireland)
Jūratė Macijauskienė	Section President (Lithuania)
Marianne van Iersel	Section President-Elect (Netherlands)
Maria S. Nuotio	Member (Finland)
Helgi Kolk	Member (Estonia)
Eva Topinková	Member (Czech Republic)
Didier Schoevaerdts	Member (Belgium)
Maria Victoria Farré Mercadé	Member (Spain)
Christophe Graf	Member (Switzerland)
Santiago Cotobal Rodeles	Member, European Junior Doctors (Spain)
Mark Anthony Vassallo	Member (Malta)
Michael Vassallo	Member (UK)
Dieter Lütje	Member (Germany)

### Foundation and prior consensus

From the outset, there was unanimous agreement among the Committee members that the 2025 ETR should build directly upon the 2019 European Postgraduate Curriculum in Geriatric Medicine [16], which had already been integrated into the 2020 ETR. Developed through a modified Delphi process under the auspices of the UEMS-GMS, EuGMS, and EAMA, the 2019 curriculum constituted the first major European consensus defining the theoretical knowledge base and competency framework of the specialty. There was also unanimous agreement to maintain a recommended minimum five-year duration of postgraduate training in Geriatric Medicine, despite the four-year minimum set by Directive 2005/36/EC. In addition, the 2025 ETR continues to promote inclusive, non-ageist language that supports a respectful, person-centred approach to ageing [17].

### Educational framework and alignment

The overarching goal of the 2025 ETR revision was to further align training standards with contemporary principles of Competency-Based Medical Education (CBME). CBME is a learner-centred approach that emphasises the attainment of measurable abilities rather than time-based progression, ensuring that advancement through specialist training is determined by the demonstration of defined competencies. Accordingly, the Committee sought to update the ETR framework by further expanding and integrating the Entrustable Professional Activities (EPAs) developed in the 2020 ETR (discrete, observable professional tasks that trainees must be able to perform independently) as the operational bridge between theoretical competencies and clinical practice [18, 19]. Whilst recognising the heterogeneity of EPA implementation across European countries, the Committee reached consensus that CGA, the defining hallmark of Geriatric Medicine, should serve as the “core or stem” upon which most clinical EPAs are constructed.

### Review and consultation

The revision maintained the established three-part structure of UEMS ETR documents, comprising Training Requirements for Trainees, Training Requirements for Trainers, and Training Requirements for Training Institutions. The content of training and learning outcomes was comprehensively organised to encompass theoretical knowledge, practical and clinical skills, and professionalism, ensuring a balanced and competency-oriented framework for postgraduate education. The organisation of the training section outlines the overall structure and process of specialist education, including the schedule of training, use of logbooks or training portfolios, and further integration of EPAs (building on the 2020 ETR) within the assessment and evaluation framework. It also details the application of assessment tools and processes, remedial actions, end-of-training assessments, and governance standards, providing a coherent and transparent structure for quality assurance. The drafting process of the 2025 ETR involved multiple iterative cycles of internal review within the Committee.

### External endorsement

Following internal approval by the UEMS-GMS, the revised ETR was circulated to key European and international partner organisations for formal endorsement. This process reflected the collaborative and multidisciplinary nature of the specialty, ensuring alignment with leading academic and professional bodies. As such, written endorsements were received from EAMA, EuGMS, the International Association of Gerontology and Geriatrics (IAGG), and the European Interdisciplinary Council on Ageing (EICA) (see endorsement dates in Table 2).

**Table 2:** Timeline of the 2025 revision of the European Training Requirements (ETR) for the Specialty of Geriatric Medicine

Date	Event
October 17, 2020	Previous ETR for Geriatric Medicine approved by the UEMS Council (reference point for the current revision cycle)
September 21, 2024	Initiation of the 2025 revision at the UEMS-GMS meeting in Valencia, Spain
October 23, 2024	First meeting of the UEMS-GMS ETR Review Committee
February 14, 2025	Completion of the internal drafting phase
April 9, 2025	Draft approved by UEMS-GMS at the Spring Online Meeting
May 8, 2025	Endorsement received from the European Academy for Medicine of Ageing (EAMA)
May 9, 2025	Endorsement received from the European Geriatric Medicine Society (EuGMS)
May 30, 2025	Endorsement received from the International Association of Gerontology and Geriatrics (IAGG)
June 1, 2025	Submission of endorsed document to the UEMS Secretariat
July 19, 2025	Conclusion of the formal UEMS review period
August 19, 2025	Incorporation of minor revisions following reviewer feedback
September 15, 2025	Endorsement received from the European Interdisciplinary Council on Ageing (EICA)
September 27, 2025	Version for consideration at the Autumn Meeting of the UEMS Council, approved by the UEMS-GMS
October 17, 2025	Finalised ETR approved by unanimity at the UEMS Advisory Board Meeting, Tbilisi, Georgia
October 18, 2025	Finalised ETR approved by unanimity at the UEMS Council Meeting, Tbilisi, Georgia
October 24, 2025	ETR 2025 published on the UEMS website (https://www.uems.eu/european-training-requirements): Geriatric Medicine (2025/29)

### Timeline of the 2025 ETR revision

The revision of the ETR for the Specialty of Geriatric Medicine followed a structured process that combined systematic drafting, stakeholder consultation, and formal review within the UEMS-GMS. The process began in September 2024 and culminated in the formal approval of the finalised ETR at the UEMS Autumn Council Meeting in Tbilisi, Georgia, on October 17–18, 2025. The ETR 2025 was subsequently published on the 24 October on the UEMS website (https://www.uems.eu/european-training-requirements). A summary of the key milestones is presented in Table 2.

## Innovation highlights

**Fig. 2:**
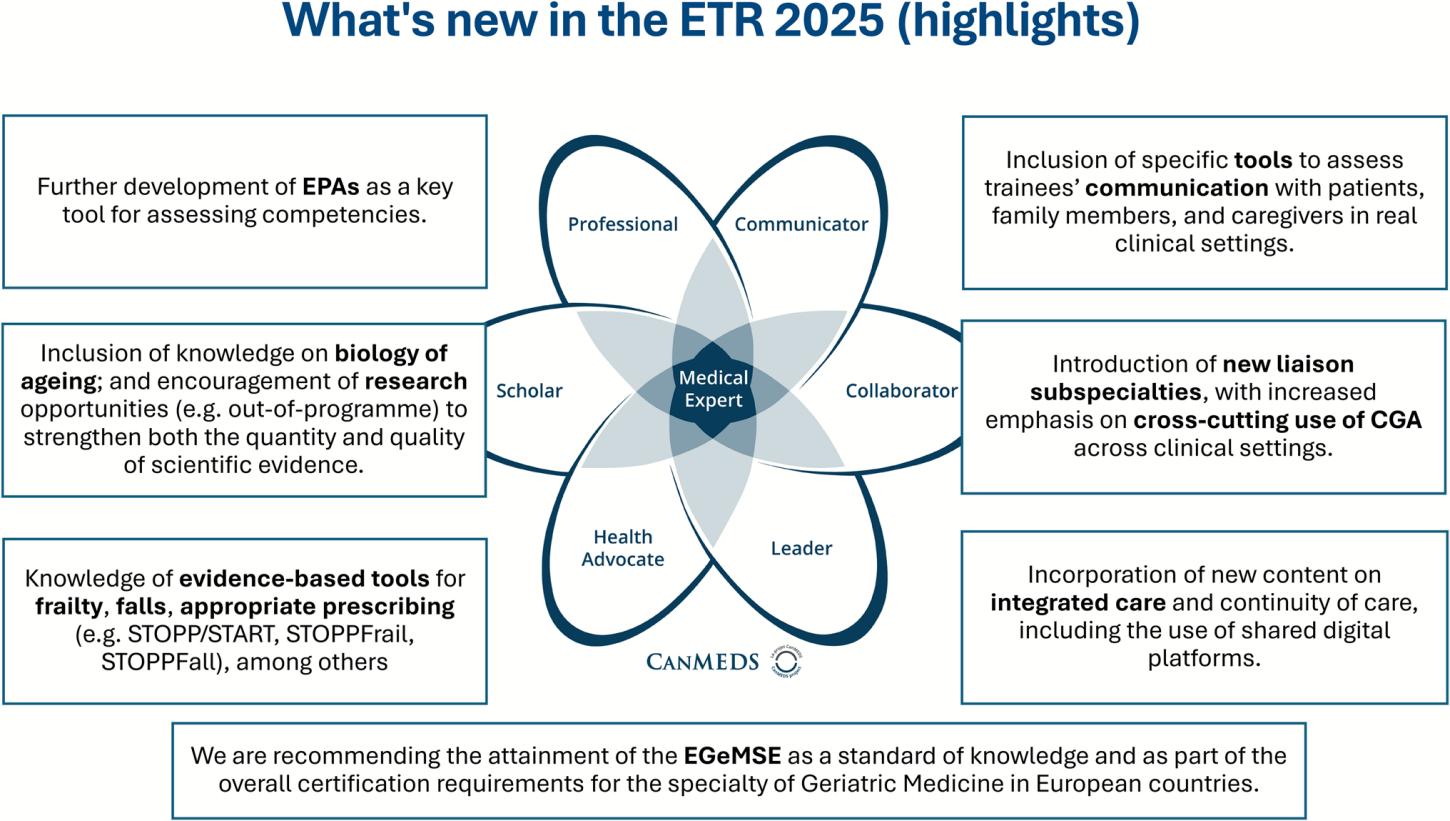
Innovation highlights of the 2025 revision of the ETR for the Specialty of Geriatric Medicine (https://www.uems.eu/european-training-requirements)

Building on the 2020 edition, the 2025 revision of the ETR for the Specialty of Geriatric Medicine (https://www.uems.eu/european-training-requirements) introduces substantial conceptual, structural, and pedagogical refinements that reflect the continued maturation of the discipline and advances in CBME **(**Fig. 2**)**.

### Clarified definitions, scope, and evolution of the specialty

The 2025 ETR introduces a new section on the Specialty of Geriatric Medicine, which modernises and clarifies key definitions whilst recognising the evolving scope of the discipline across Europe. The updated definitions of Geriatric Medicine and the Geriatrician [6], already outlined in the Introduction, reaffirm the specialty’s holistic focus on older adults with frailty and multimorbidity and its foundation in CGA. Importantly, the revision explicitly states that Geriatric Medicine is *not age-defined*; whilst most patients are aged 65 years and older, geriatric syndromes are most prevalent amongst those aged 80 years and above. The ETR underscores the versatility of Geriatric Medicine across the continuum of healthcare settings, reflecting its capacity to respond to the healthcare needs of all older adults. The specialty spans acute, rehabilitative, outpatient, community, and long-term care. Geriatricians play a pivotal role in coordinating and integrating multidisciplinary services, increasingly collaborating through a network of liaison subspecialties, each working in partnership with its corresponding non-geriatric specialty; for example, orthogeriatrics with Orthopaedics and Traumatology, oncogeriatrics with Medical Oncology, psychogeriatrics with Psychiatry, perioperative medicine for older people (POPS) with Anaesthesiology, Pain Medicine, and Surgery, geriatric rehabilitation with Physical and Rehabilitation Medicine, geriatric emergency medicine (GEM) with Emergency Medicine, cardiogeriatrics with Cardiology, neurogeriatrics with Neurology, and nephrogeriatrics with Nephrology, amongst others that continue to emerge.

### Expanded knowledge base

The 2025 ETR significantly broadens the knowledge base of specialist training to reflect advances in biomedical, clinical, and social sciences. The updated syllabus places greater emphasis on understanding the molecular and cellular mechanisms of ageing, highlighting the conceptual distinction between chronological and biological age, and the evidence-based hallmarks of accelerated biological ageing, reflecting the substantial growth of this field over the past 5 years [20].

In the area of multimorbidity and geriatric syndromes, previously established thematic areas have been expanded. For example, beyond a basic understanding of the concept of frailty, there is now a requirement to demonstrate knowledge of the advantages and limitations of different frailty identification tools [21]. In the area of cognitive disorders and dementia, there has been explicit mention of the latest evidence on prevention [22, 23], as well as the collaborative diagnostic roles of Geriatric Medicine, Neuroradiology, and Laboratory Medicine. Similarly, in the area of falls, trainees are now expected to be familiar with more detailed and evidence-based guidelines on falls prevention and management [24]. In addition, new explicit knowledge requirements have been introduced in areas such as hospital-associated deconditioning [25], loneliness, and social vulnerability, which are recognised as key contributors to frailty and functional decline in older adults.

In the area of drug therapy, whilst the 2020 ETR provided a non-exhaustive list of drugs frequently prescribed to older patients that should be regularly reviewed, the 2025 ETR introduces significant updates. These include the application of validated prescribing and assessment tools, notably STOPP/START v3 [26], STOPPFrail [27, 28], and STOPPFall [29], to promote safer and evidence-based medication management.

The 2025 ETR substantially expands the domains of integrated care and specific clinical pathways and health promotion, aligning them with contemporary international frameworks such as the WHO Integrated Care for Older People (ICOPE) [30]. Trainees are now expected to demonstrate an understanding of integrated and person-centred care systems that bridge the traditional boundaries between health and social services. This includes identifying and coordinating across multiple levels of care whilst ensuring continuity through effective care transitions and multidisciplinary collaboration. The ETR highlights the importance of leveraging digital record-sharing platforms and recognising national legislative and regulatory frameworks that support integration. Health promotion has also been reframed to reflect a lifecourse and functional capacity perspective, encompassing preventive strategies, risk reduction, and the creation of age-friendly environments.

In addition, in the area of Evidence-Based Medicine, the 2025 ETR explicitly calls for continuous knowledge generation and encourages the uptake of research opportunities (e.g. out-of-programme experiences) to strengthen both the quantity and quality of scientific evidence in the field.

### Refined competency levels

**Fig. 3:**
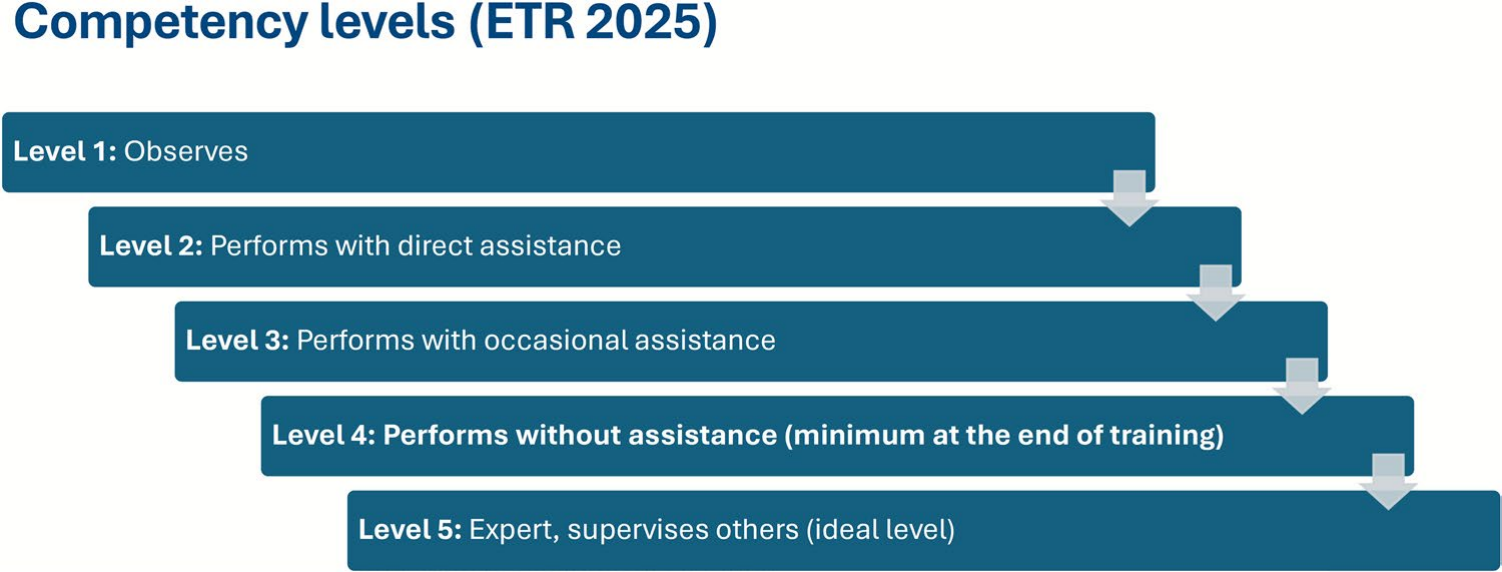
Competency levels in the 2025 ETR for the Specialty of Geriatric Medicine

The competency framework has been streamlined to five levels, providing greater clarity and alignment with the principles of CBME. The new Level 5 (“Can expertly perform without assistance”) represents the ideal standard of independent expert practice expected of a newly qualified specialist, combining professional autonomy with supervisory and teaching capability. Level 4, denoting independence, is defined as the minimum requirement for completion of specialist training (Fig. 3). The ETR 2025 specifies that a clear, objective, and nationally agreed procedure must be established to determine a trainee’s progression from Level 3 (“Performs with occasional assistance”) to Level 4, ensuring transparency and consistency in competence-based progression.

### Deeper integration and formalisation of EPAs

Whilst the 2020 ETR introduced EPAs at a conceptual level, the 2025 revision operationalises them within a fully developed competency-based education framework. There remains considerable variation in the implementation of EPAs across European Geriatric Medicine training programmes, ranging from full integration to limited or no adoption. Despite these national differences, CGA is recognised as the common foundation underpinning most clinical EPAs, adapted to diverse healthcare and educational contexts. The 2025 ETR explicitly states that CGA serves as the foundational “core or stem” from which other clinical EPAs branch out [31], underscoring its dual role as both a clinical methodology and an educational framework. The updated ETR provides a non-exhaustive list of 15 clinical and 2 non-clinical EPAs, which may be adopted or adapted to meet national training requirements. Four exemplar EPAs, three clinical (CGA; Comprehensive Medication Review and Optimisation; and Prevention, Assessment and Management of Delirium) and one non-clinical (Teaching Geriatric Medicine to Undergraduate Medical Students), are described in full detail (see Table 3 for full detail on the CGA EPA). Each is mapped to specific domains of knowledge, skills, attitudes, and behaviours, with clearly defined assessment indicators and entrustment criteria, illustrating how EPAs integrate theoretical learning with workplace-based performance and progressive professional autonomy.

**Table 3:** EPA example: Comprehensive Geriatric Assessment (CGA)

Description	Conducting a comprehensive assessment of older adults across medical, psychological, functional, and social domains to develop a tailored, integrated care plan that addresses individual needs in any care setting
Knowledge	Evidence-based guidelines
	Knowledge of the care setting
	Indications for CGA
	Components of a CGA
	Professionals involved in a CGA
	Standardised assessment tools
Skills	Thorough history directly from the patient
	Collection of collateral history when required
	Conducting a comprehensive physical examination
	Performing physical, cognitive, and functional assessments
	Carrying out a psychosocial evaluation
	Synthesising and prioritising findings
	Creating a personalised care plan
	Consulting with other specialists as appropriate
	Accurate documentation
	Effective presentation
Attitudes and behaviours	Collaboration: working effectively with multidisciplinary teams to conduct assessments and develop coordinated care plans
	Clearly and empathetically sharing assessment findings and care plans with patients and their caregivers
	Professionalism: providing ethical, patient-centred care that upholds the values, preferences, confidentiality, and dignity of older adults
Information to assess progress (examples)	Real-time performance evaluation: direct observation during CGA activities
	Focussed clinical assessment: Mini-Clinical Evaluation Exercise (Mini-CEX) for evaluating specific skills and decision-making
	Clinical reasoning review: case-based discussions to assess application of CGA principles
	Team-based feedback: input from multidisciplinary teams on collaboration and contributions
	Outcome monitoring: evaluation of patient outcomes resulting from CGA-informed care plans
When is unsupervised practice expected?	Unsupervised practice is expected at competency Level 4 (see Competencies section)
Basis for formal entrustment decisions	Entrustment is based on satisfactory completion of a variety of assessment tools (see Assessment Tools section)

### Enhanced assessment and quality assurance

The assessment and quality management framework in the 2025 ETR has been modernised and expanded to ensure transparency, consistency, and alignment with contemporary principles of CBME. A broader and more structured set of assessment tools is now recommended to evaluate trainees across multiple domains of knowledge, skills, and professional behaviours. These include the Mini-Clinical Evaluation Exercise (Mini-CEX) and Direct Observation of Procedural Skills (DOPS), which assess clinical performance in real time; Case-Based Discussions (CBD), designed to evaluate clinical reasoning and decision-making; and Multi-Source Feedback (MSF), or 360-degree assessment, which collects feedback from colleagues across disciplines to provide a comprehensive view of performance. The Objective Structured Clinical Examination (OSCE) is also highlighted as a structured and standardised method for assessing clinical competence. It consists of a series of timed stations in which candidates perform specific tasks (such as history taking, physical examination, communication, or interpretation of clinical data) under direct observation, with performance measured using objective criteria. In addition, completion of accredited leadership and procedural courses (e.g. in echocardiography) is encouraged to strengthen professional breadth and foster transferable skills relevant to multidisciplinary practice.

**Fig. 4:**
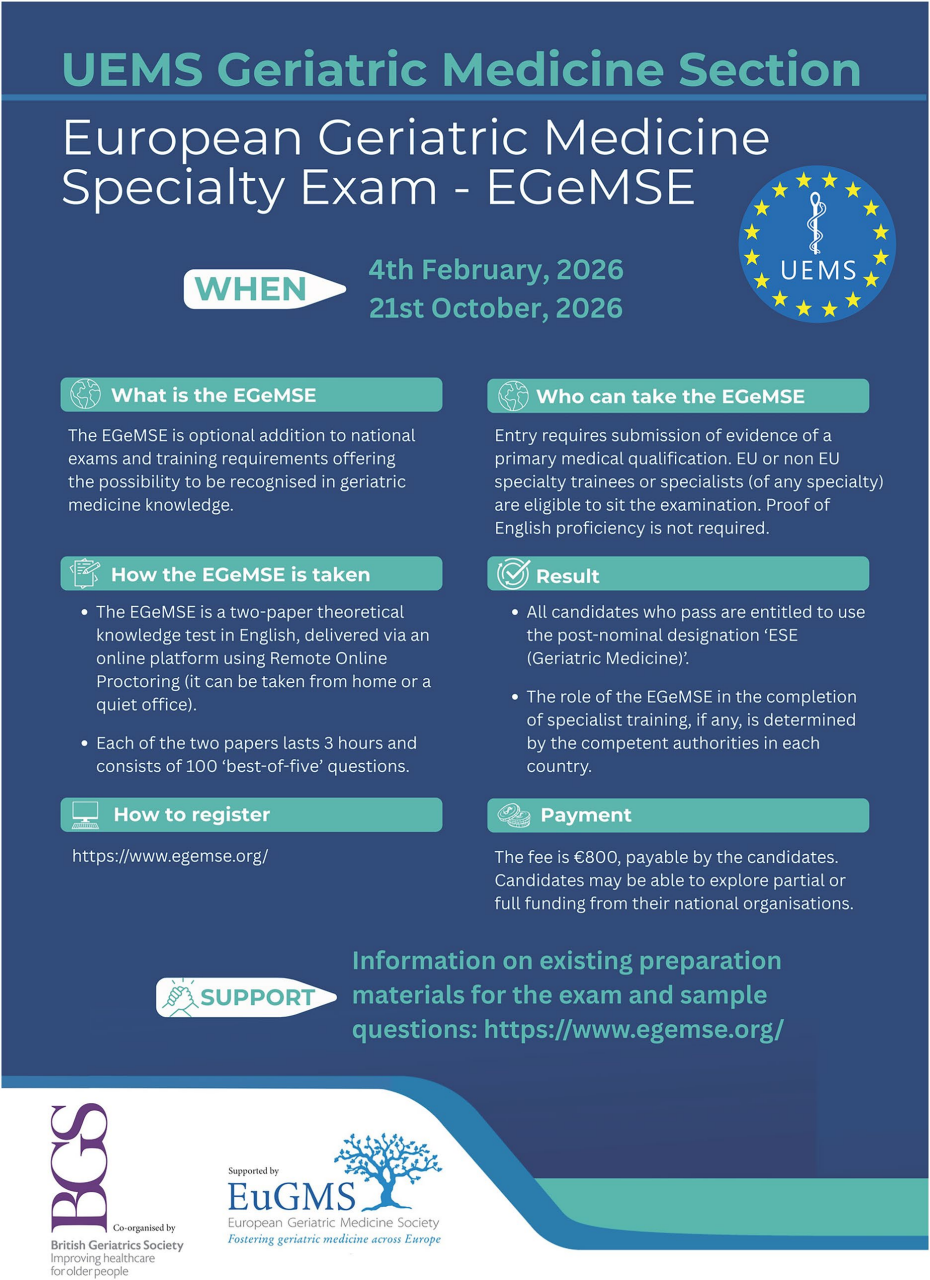
The European Geriatric Medicine Specialty Exam (EGeMSE). Further details are available at https://www.egemse.org/ and https://www.uemsgeriatricmedicine.org/

Importantly, the European Geriatric Medicine Specialty Exam (EGeMSE) (Fig. 4) is now formally endorsed by the 2025 ETR as a pan-European benchmark of theoretical knowledge, supporting harmonised certification standards and promoting comparability of training outcomes across Member States (Fig. 4). The inaugural EGeMSE took place on April 23, 2025, with the first cohort of candidates from across Europe successfully completing the online examination. The exam is organised by the UEMS-GMS, in cooperation with the British Geriatrics Society (BGS) and the Federation of Royal Colleges of Physicians of the UK, and follows the same model as other European specialty examinations, such as the European Specialty Examination in Gastroenterology & Hepatology (ESEGH) and the European Specialty Examination in Nephrology (ESENeph). The EGeMSE consists of two three-hour online papers in English, each comprising 100 best-of-five multiple-choice questions, and successful candidates are awarded the post-nominal title ESE (Geriatric Medicine) [32]. The examination is open to both EU and non-EU doctors in training or qualified specialists and is designed to complement rather than replace existing national knowledge-based assessments. In some countries (e.g. Switzerland and Ireland at the time of writing), the EGeMSE has already been recognised by national competent authorities as a valid alternative or supplement to domestic knowledge-based examinations, whilst in others it provides a valuable additional option where such assessments are not yet established.

### Other improvements

Additional refinements to the 2025 ETR resulted from the round of minor revisions (July–August 2025), informed by feedback from the central UEMS ETR Review Committee and other UEMS bodies. Key updates included the addition of a glossary and list of abbreviations, clarification of the distinction between syllabus and curriculum, and clear differentiation between formative and summative assessments within CBME. The 2025 ETR places greater emphasis on interdisciplinary collaboration across diagnostic and therapeutic domains. New content strengthens cooperation with radiologists through an expanded section on imaging interpretation and provides clearer guidance on dementia-related imaging, whilst underscoring the pivotal role of neuroradiologists. Collaboration with laboratory medicine specialists is reinforced through updated guidance on the appropriate use of laboratory and point-of-care testing. The medication review EPA now underscores the importance of close collaboration with clinical pharmacologists and pharmacists to ensure safe and evidence-based prescribing. Cooperation with Physical and Rehabilitation Medicine (PRM) specialists has also been recognised through the encouragement of mutual consultation and explicit reference to the WHO International Classification of Functioning, Disability and Health (ICF) model [33], promoting integrated, function-oriented care.

## Discussion

The 2025 ETR for the Speciality of Geriatric Medicine represents an evolving shift from a largely structure-oriented curriculum toward an outcomes-driven, competency-based framework. The revised ETR consolidates recent scientific, educational, and policy developments into a coherent standard that defines not only what geriatricians must know, but how they must perform, in real clinical contexts and within multidisciplinary teams. In addition, it delineates the standard requirements for trainers and training institutions.

The revision process itself represents a key strength. It builds on prior consensus (the 2019 curriculum and 2020 ETR), adheres to UEMS procedures, and, for the first time, received endorsement from four major European and international organisations (EAMA, EuGMS, IAGG, and EICA). The active engagement of European Junior Doctors and a broad range of national representatives ensured relevance across diverse healthcare and educational systems, whilst minor revisions following the UEMS review further strengthened interdisciplinarity and alignment with contemporary practice.

The 2025 ETR encourages the adoption or adaptation of its contents rather than uniform prescription, an approach designed to enhance feasibility and uptake. Indeed, there remains substantial variation in training and recognition across Europe, underscoring the continuing need for initiatives to promote shared educational goals and to support the development of Geriatric Medicine where it is still emerging [34]. Whilst it is recognised that the ETR sets a relatively high benchmark in countries where Geriatric Medicine is less well established, it aspires to a “race to the top” rather than a “race to the bottom,” fostering upward convergence through shared standards of excellence and mutual learning. Although progress will inevitably be gradual, broader awareness of the ETR’s principles and content can serve as a catalyst for this collective advancement. In addition, emphasising CGA as the “core or stem” EPA may help address the recently identified need for more coordinated research in CGA education and training, thereby consolidating the evidence base and supporting the development of innovative, high-quality healthcare systems capable of responding to the challenges of our ageing populations [35].

Implementation will require deliberate investment. Faculty development is essential to ensure consistent entrustment decisions, reliable use of assessment tools, and meaningful feedback. Training portfolios and digital solutions will need to support EPA tracking without adding undue administrative burden. Because recognition of Geriatric Medicine is not yet uniform across Europe, continued policy engagement is necessary, both to advance specialty recognition in jurisdictions where it is absent or where subspecialty status persists, and to align national certification pathways with the shared European standard. In this context, the EGeMSE can serve as a pragmatic lever for comparability of knowledge, whilst respecting national regulatory differences; importantly, however, it represents a standard of knowledge rather than of skills or competencies.

In sum, the 2025 ETR revision promotes inclusive, contemporary, and evidence-based terminology and content in Geriatric Medicine, providing a comprehensive, forward-looking framework that advances harmonisation and excellence fit for purpose for the next 5 years. It also highlights new opportunities for collaboration with other specialties to optimise care for older adults across all healthcare settings. We would like to thank everyone involved in the 2025 ETR revision process. The UEMS-GMS remains open to dialogue and is committed to fostering collaboration with all relevant specialties and stakeholders to advance the care of older adults across Europe and beyond.

## Data Availability

Not applicable.

## Declarations

### Approved by the UEMS Council

*Tbilisi, Georgia, 17–18 October 2025.* Available from: https://www.uems.eu/european-training-requirements. (The adopted ETRs, Geriatric Medicine, 2025/29).

**LINK to ETR 2025**: https://www.uems.eu/european-training-requirements: Geriatric Medicine (2025/29).
